# Problem Gambling ‘Fuelled on the Fly’

**DOI:** 10.3390/ijerph18168607

**Published:** 2021-08-14

**Authors:** Joseph Teal, Petko Kusev, Renata Heilman, Rose Martin, Alessia Passanisi, Ugo Pace

**Affiliations:** 1 Behavioural Research Centre, Huddersfield Business School, The University of Huddersfield, Huddersfield HD1 3DH, UK; joseph.teal@hud.ac.uk; 2 Department of Psychology, Babeş-Bolyai University, 400015 Cluj-Napoca, Romania; renataheilman@psychology.ro; 3 Department of People and Organisations, Surrey Business School, University of Surrey, Guildford GU2 7XH, UK; r.k.martin@surrey.ac.uk; 4 Faculty of Human and Social Sciences, UKE—Kore University of Enna, Cittadella Universitaria, 94100 Enna, Italy; alessia.passanisi@unikore.it (A.P.); ugo.pace@unikore.it (U.P.)

**Keywords:** problem gambling, risky decision-making, anchoring on recent events, construction of preferences, dynamic experience of events in time series

## Abstract

Problem gambling is a gambling disorder often described as continued gambling in the face of increasing losses. In this article, we explored problem gambling behaviour and its psychological determinants. We considered the assumption of stability in risky preferences, anticipated by both normative and descriptive theories of decision making, as well as recent evidence that risk preferences are in fact ‘constructed on the fly’ during risk elicitation. Accordingly, we argue that problem gambling is a multifaceted disorder, which is ‘fueled on the fly’ by a wide range of contextual and non-contextual influences, including individual differences in personality traits, hormonal and emotional activations. We have proposed that the experience of gambling behaviour in itself is a dynamic experience of events in time series, where gamblers anchor on the most recent event—typically a small loss or rare win. This is a highly adaptive, but erroneous, decision-making mechanism, where anchoring on the most recent event alters the psychological representations of substantial and accumulated loss in the past to a representation of negligible loss. In other words, people feel better while they gamble. We conclude that problem gambling researchers and policy makers will need to employ multifaceted and holistic approaches to understand problem gambling.

## 1. Decision-Making under Risk

Research investigating decision-making under risk has been dominated by normative theories of decision-making, which have stringent expectations of human agency. Specifically, normative theories assume that ‘rational’ agents obey economic norms (e.g., possess stable and consistent preferences, which are context-independent), and evaluate choice options according to utilitarian expectations (they seek to maximise their personal utility, and minimise their personal disutility). Accordingly, in order to maximise their utility when making a decision, expected utility theory (EUT) [1] and subjective expected utility theory (SEU) [2] propose that normatively rational agents must: (1) use computational processing to transform subjective (in SEU) or objective (in EUT) values (e.g., probability and money) into a common representation of utility (expected value) for each choice option, (2) trade-off between choice options using their expected values, and choose the choice option with the highest expected value. However, whilst the normative theories provide an eloquent account of how known (EUT) and unknown (SEU) outcomes and probabilities can be transformed into expected value, empirical evidence suggests that in reality, people violate economic norms and assumptions, and are poor utility maximisers [3,4,5,6,7,8,9,10,11].

More recently, in response to the empirical evidence that people are poor normative agents, who demonstrate behavioural inconsistency with the predictions of normative theories (EUT and SEU), Kahneman and Tversky [12] and Tversky and Kahneman [13] developed prospect theory. Prospect theory—undisputedly the most influential descriptive theory in behavioural economics—describes how people typically behave when making decisions under risk and uncertainty. Accordingly, as with EUT and SEU, prospect theory assumes that people’s risk preferences are informed by computational processing. However, to account for non-normatively rational features of human cognition, prospect theory assumes that people possess non-linear probability and hypothetical value weighting functions, which facilitate non-linear transformation of object values. In particular, the probability weighting function captures people’s tendency to overweight low probability events (e.g., the likelihood of winning the national lottery), and underweight medium and high probability events (e.g., events which are almost certain; see [3]). Moreover, the hypothetical value weighting function is concave for gains and convex for losses (non-linear), as people demonstrate reduced sensitivity to gains and losses (diminishing marginal utility) as they shift away from a reference point, such as their current wealth. Furthermore, the hypothetical value weighting function is also steeper in the domain of loss than gain due to loss aversion—people feel more disutility from losses than they do utility from equivalent gains (e.g., a loss of £50 feels much worse than the pleasure derived from a gain of £50). Accordingly, when the weighting functions are considered together, prospect theory [12,13] predicts a general four-fold pattern of risk preferences: risk-seeking when there is a low probability of gaining money or when there is a high probability of losing money, and risk-aversion when there is a high probability of gaining money or a low probability of losing money [14].

Within behavioural economics research, loss aversion—the principle that losses loom larger than equivalent gains—is widely regarded as a stable feature of human cognition (see [15]). Accordingly, the loss aversion phenomenon has been generalised and applied beyond the domain of risk to explain famous non-risky behavioural phenomena, including the endowment effect [16,17] and status quo bias [18,19]. However, more recently the descriptive behavioural status of loss aversion has been called into question by researchers who have argued that empirical findings which have been attributed to loss aversion can be better explained by alternate psychological processes (see [15]). For example, ref. [20] argued that inertia (a behavioural tendency to favour the status quo over change) offers a more logical and consistent account for behavioural phenomena, including status quo bias and the endowment effect, thus rendering loss aversion superfluous. Moreover, research has demonstrated that loss aversion is not as homogeneous as once thought. Specifically, rather than being a stable feature of human cognition, the propensity for loss aversion is sensitive to methodological features of the elicitation task [21], and people’s prior experiences [22,23]. In particular, Walasek and Stewart [22] concluded that loss aversion is an artefact of experimental design, as they demonstrated that by manipulating the range of possible gains and losses which participants experienced in their experiments (and thus which are in participants’ memories), they could elicit loss aversion, loss-averse neutrality and reverse loss aversion during choice between mixed 50/50 gambles.

**Figure 1:**
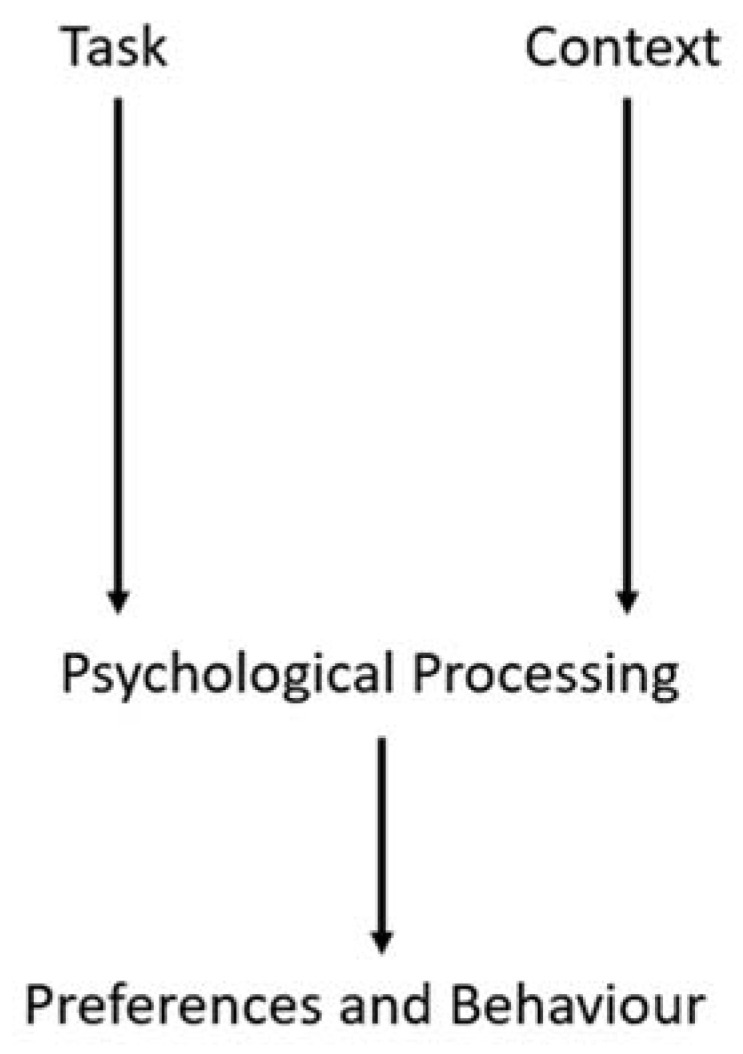
Construction of preferences ‘on the fly’.

More generally, Ert and Erev [21] and Walasek and Stewart [22] findings are consistent with contemporary behavioural economics research, which indicates that human risk preferences are not stable and consistent [14,24,25]. Instead, experimental evidence by Kusev and colleagues [14] suggests that people’s risk preferences are ‘constructed on the fly’ (see also [26]; see Figure 1) and can be informed by a range of psychological processes which are sensitive to features of the context and task [27,28,29,30,31,32,33,34]. Accordingly, in contrast to EUT, SEU and prospect theory, which all assume preference stability, as people’s preferences are constructed rather than revealed, they are unstable across different methods of elicitation [35,36]. For example, using the certainty equivalent (CE) elicitation method which Tverky and Kahneman [13] used to validate prospect theory predictions, Kusev et al. [14] demonstrated that the four-fold pattern of risk preferences is an artefact of Tversky and Kahneman’s experimental method. Specifically, they found that during repeated choice between safe (offering a sure monetary amount) and risky gambles, participants demonstrated the four-fold pattern of risk preferences only when the safe options were logarithmically (unevenly) spaced around the expected value of the risky options. In contrast, when the safe options were linearly (evenly) spaced around the expected value of the risky options, participants demonstrated a consistently two-fold pattern of risk preferences (risk-aversion in the domain of gain, and risk-seeking in the domain of loss), which were independent from probability level. Thus, a single methodological change (logarithmic to linear spacing of sure options around the expected values of risky options) had a profound influence on participants’ risky choices.

## 2. Problem Gamblers’ Risk Preferences

From an economic perspective, to decide whether and how much to gamble, people need to integrate probabilistic information (probability and reward magnitude) under conditions of risk (where this information is known—e.g., with a roulette wheel) or uncertainty (where this information is not known—e.g., sports betting) [37]. Accordingly, as prospect theory [12,13] is the most influential descriptive theory of decision-making under risk and uncertainty, and caters for individual difference in probability and value weighting, it has been used as a tool to explore problem gambling. Problem gambling is a general term used widely in research to encompass compulsive gambling, addictive gambling, and gambling disorder [38]. In the Diagnostic and Statistical Manual of Mental Disorders (DSM-5), gambling disorder is regarded as a behavioural addiction which has a prevalence of approximately 0.2% to 0.3% in the general population, and is associated with ‘continued gambling in the face of mounting losses, often at the detriment of financial, social and occupational obligations’ [37] (p. 1).

In agreement with the evidence that loss aversion is not homogeneous, it has been suggested that individual differences in propensity for loss aversion can explain the excessing risk-seeking behaviour which characterises problem gambling [39]. This hypothesis, that problem gamblers exhibit reduced loss aversion relative to healthy controls, has been examined in a number of studies which have used experimental methods from behavioural economics [39,40,41]. However, the results are mixed, with some studies finding that problem gamblers are less loss-averse when compared with healthy controls [40,42,43], and others reporting no significant difference in loss aversion between problem gamblers and healthy controls [39,41]. One plausible explanation for the mixed findings across studies is that problem gamblers’ propensity for loss aversion increases throughout their clinical treatment [40]. Specifically, researchers [40,42] found evidence of loss aversion because their problem gambler participants had either no treatment or little treatment, whilst Takeuchi and colleagues [41] did not find evidence for loss aversion as their problem gambling participants had already undergone clinical treatment. This proposal is consistent with evidence from Giorgetta and colleagues [44], who found that problem gamblers in the later stages of clinical treatment (>18 months) were more loss-averse than healthy controls and problem gamblers in the early stages of clinical treatment (<6 months).

However, it is also possible that problem gambling might be related to increased distortion or elevation of prospect theory’s [12,13] probability weighting function [39]. In particular, with regard to distortion of the probability weighting function, as gambles typically offer small probability gains, problem gambling might be related to problem gamblers overweighting small probabilities more relative to healthy controls [39,45]. Although, with regard to elevation of the probability weighting function, as problem gamblers demonstrate risky behaviour outside the domain of gambling [46], it is possible that they overweight probabilities across the entire probability range, leading to a generally exaggerated attraction towards risky events [39,45].

In order to address these assumptions, experimental methods from behavioural economics have been used to explore whether problem gambling is caused by distortion or elevation of the probability weighting function. For example, Ligneul and colleagues [45] used the CE procedure to measure the probability weighting function for two groups of participants (problem gamblers and healthy controls). Accordingly, participants had to make a series of choices between a risky gamble offering a probability to win an amount of money, or an amount of money with certainty (a sure amount). Ligneul et al. [45] predicted that if problem gamblers suffered from probability distortion (increased distortion of the probability weighting function for low probability events) then they would demonstrate increased preference for the risky choice confined to small probability events. In contrast, they predicted that if problem gamblers suffered from elevation of the entire probability weighting function, then they would have a preference for risk independent of probability. Their results revealed that the control group replicated prospect theory’s probability weighting function (overweighting of low probability events and underweighting of medium and high probability events), whilst the problem gambling group had an elevated probability weighting function. In other words, problem gamblers had a globally exaggerated attraction to the risky gambles, which is independent of probability. However, more recently, Ring et al. [39] found that in the domain of gain, relative to healthy controls, problem gamblers demonstrated more distortion in their probability weighting, were less sensitive to probability level and were more risk seeking. In contrast, in the domain of loss, Ring et al. [39] found no significant difference in the risk attitudes of problem gamblers and controls.

Whilst variance in probability and value weighting parameters of heathy agents and problem gamblers can help to explain why these groups differ in their propensity to make risky choices, we believe that problem gambling research would benefit from exploring contextual triggers of gambling behaviour. In particular, in light of evidence that people’s risk preferences are context dependent [14,47], we speculate that problem gambling might be an unstable disorder partially driven by contextual factors. Specifically, following Kusev and colleagues’ perspective that risk preferences are ‘constructed on the fly’ [14], we believe it is plausible that problem gambling is ‘fuelled on the fly’ by a variety of contextual factors, which increase or decrease the likelihood that a problem gambler will engage in gambling behaviour (e.g., task-related factors which might trigger a problem gambler’s enhanced risk-seeking tendencies). To our knowledge, the influence of contextual and task-related factors (i.e., methodological features) has not been explicitly explored within problem gambling research, but could contribute towards explaining why problem gamblers can struggle with addiction to particular gambling activities [48]. Accordingly, one research opportunity for problem gambling researchers is to examine the stability of problem gamblers’ risky preferences across a sample of elicitation methods used in research exploring general risk preferences (see [36]), and also across different types of gambling methods (e.g., slot machines, roulette wheels).

Our ‘fuelled on the fly’ problem gambling proposal is consistent with the view that problem gambling is primarily caused by heuristics and biases [49,50], triggered by contextual features (e.g., roulette tables which show gamblers the outcome of previous spins) [51]. Accordingly, some gambling products are configured to exploit decision-making biases. For instance, in a recent study Newall and colleagues [52] found that custom sports bets (where gamblers are allowed to create their own custom bets) are particularly popular among problem gamblers, and give them a greater illusion of control relative to a non-problem gambler sample. Moreover, custom sports bets can also be configured to exploit the representativeness heuristic [53].

## 3. Triggers of Gambling Behaviour: The Role of Personality, Hormonal and Emotional Activations

Independently of contextual factors, research investigating human preferences under conditions of risk and uncertainty has demonstrated that people’s behaviour is also influenced by a large number of biological and psychological factors not accounted for in classic economic theory for a review (see [54]). Accordingly, in congruence with our earlier proposal that problem gambling is ‘fuelled on the fly’ by a variety of contextual factors, we also assume that people’s likelihood of engaging in gambling activities (and subsequently of becoming a problem gambler) is, to a large extent, determined by individual differences. In other words, we speculate that problem gambling is a multifaceted disorder, which is fuelled by a wide range of contextual (discussed in the previous section) and non-contextual influences, including individual differences in biological (hormonal activations) and psychological (personality traits, and emotional activations) factors. Given the above, in this section we will discuss evidence that individual differences in the aforementioned factors can trigger people’s risky preferences and behaviour, and discuss their relevance to problem gambling.

### 3.1. The Role of Personality

Research investigating personality has been dominated by ‘trait theories’ which generally assume that people’s personalities are internal, relatively enduring and composed of a conglomerate of individual traits. For example, scholars [55,56,57] have developed the five-factor model of personality (commonly known as ‘the big five’), according to which personality is organized across five core dimensions. These core dimensions are: (1) neuroticism, (2) extraversion, (3) openness to experience, (4) conscientiousness and (5) agreeableness. Since its development, the five-factor model has become the most dominant trait theory, and has been used to predict individual differences in a variety of settings [58].

Given the above, it is not surprising that researchers investigating the influence of personality on risky behaviour have revealed that people’s risk preferences for choice between gambles, and in everyday life, are associated with the big five personality factors for a review (see [59]). Specifically, the factors of conscientiousness and neuroticism are frequently associated with risk-averse preferences [59], and extraversion is most consistently associated with risk-seeking preferences [59,60]. For example, in everyday life, extraversion is a predictor of a variety of risky behaviours, including participation in risky sports [61], involvement in traffic collisions [62], smoking [63] and financial debt [64]. Furthermore, the influence of the big five personality factors has also been explored within the context of problem gambling. For example, evidence suggests that relative to non-problem gamblers, problem gamblers score higher on the neuroticism factor and lower on the conscientiousness factor [41,65]. In particular, Brunborg et al. [66] demonstrated that the association between neuroticism and conscientiousness increases with the severity of the problem gambling (low, moderate, severe).

The rank-order and absolute (or mean level) stability of the big five personality traits have been widely investigated in personality research (see [67,68,69]). Studies which have examined the stability of the big five personality traits using test–retest method have found substantial correlations, and overall consistency of individual differences over time exemplified in the rank-order stability (see [69]). Moreover, consistent with Caspi et al.’s [67] cumulative continuity principle, the rank-order stability of the big five personality traits increases with age [70]. Although, there is less certainty about the age at which rank-order stability reaches its peak (see [68,69]). For example, in contrast to McCrae and Costa [71] who proposed that personality is essentially fixed by the age of 30, in a meta-analysis Roberts and Delvecchio [72] reported that test–retest correlations continue to improve after age 30, and peak between ages 50 and 70. Results from studies which have examined the absolute stability (mean-level differences in personality overtime) are generally summarized by Caspi et al.’s [67] maturity principle of personality development, which suggests that mean-level changes in personality represent psychological maturity and adjustment to society. In particular, research indicates that as people age, average levels of the traits’ conscientiousness and agreeableness increase (see [69]), possibly because these traits enable people to become more productive contributors to society [67,68]. Moreover, average level of neuroticism decreases with age (see [64]). Although, the relationship between aging and the traits of extraversion and openness to experience is less clear, with some studies indicating that average levels of these personality traits increase over time [68], and other studies finding that average levels decrease over time [73]. Furthermore, in contrast to the trait theories of personality, in his social-cognitive theory, Bandura hypothesises two-way bidirectional interactions between personal factors (cognitive, affective, and biological events) and social environments (triadic reciprocal causation) [74]. In other words, behaviour influences and is influenced by personal factors and social environments.

### 3.2. The Role of Hormonal Activations

Over a period of around a week, Coates and Herberg [75] examined the impact of market volatility (characterised by uncertainty) on the cortisol (the stress hormone) levels of day-traders based in the City of London. Their results demonstrated that as market volatility increased, participants’ mean daily cortisol levels increased by 68%. Moreover, as a result of anticipated challenges, variance in return and increased sustained effort, participant’s cortisol levels were often higher when measured in the afternoon relative to when measured in the morning. Increased cortisol can have a variety of cognitive and behavioural effects [54] (for a review, see [75]). Specifically, when cortisol is increased to acute levels, it is associated with increased motivation and sensation seeking [76], focused attention and enhanced retrieval of important memories. However, when raised to chronic levels and over a longer period of time, cortisol impairs executive control and increases negative feelings, such as helpless and anxiety [77].

Accordingly, Kandasamy et al. [78] hypothesised that acute exposure to increased cortisol would, at most, modestly increase risk-seeking behaviour (although, most likely have no-significant effect at all), whereas chronic exposure to increased cortisol would significantly increase risk-averse behaviour. To explore this hypothesis, Kandasamy and colleagues used a randomised, double blind, placebo, cross-over study in which participants’ financial risky choice preferences were measured using computerised tasks (lottery choices) under control conditions (with placebo), under conditions of acute exposure to cortisol and under conditions of chronic exposure to cortisol. Accordingly, over a period of eight days, participants were administered with either a placebo or hydrocortisone (a pharmaceutical form of cortisol) to raise their cortisol to either acute or chronic levels. In congruence with their hypothesis, Kandasamy et al.’s results revealed that participants’ financial risk preferences were not significantly influenced by acute exposure to cortisol. However, when exposed to chronically raised cortisol, participants were more risk-averse and preferred safer lotteries with lower expected value and lower variance in their expected value. Consistent with research which argues that people’s risk preferences are ‘constructed on the fly’ [14,24], Kandasamy and colleague’s results indicate that their participants’ risky preferences were dynamic and not determined by stable and consistent psychoeconomic functions.

Within research investigating problem gambling, it has been revealed that problem gamblers, relative to recreational gamblers, demonstrate a blunted cortisol response when watching gambling-related videos [79]. When considered alongside evidence that higher (chronic) cortisol levels are associated with increased risk-aversion, this suggests that problem gamblers’ blunted cortisol response could explain why they are generally more risk-seeking than the healthy population. Moreover, it has also been argued that disruptions to the diurnal dynamics of cortisol secretion are associated with problem gambling. Specifically, in the healthy population, secretion of cortisol usually peaks within an hour of awakening, and then reduces from morning to the evening (diurnal fall). However, in a recent study, Buchanan et al. [80] argued that relative to a control group, a group of non-treated problem gamblers would demonstrate a blunted cortisol awakening response and a flatter diurnal fall. Accordingly, to test their hypothesis, Buchanan and colleagues experimentally examined how the different diurnal cortisol profile of non-treated problem gamblers and a healthy control group influenced their risky preferences in gamble-type tasks (the Cups Task and Columbia Card Task). In congruence with their prediction, Buchanan et al.’s. results revealed that problem gamblers had altered cortisol dynamics, including a blunted awakening cortisol response throughout the morning and a flatter diurnal fall from morning to evening. Furthermore, the results also revealed that in the both types of risky choice task (the Cups Task and Columbia Card Task), problem gamblers were more risk-seeking than healthy controls. Moreover, Buchanan et al. also found that for the problem gambling group, lower cortisol was associated with more risk-taking.

However, cortisol is not the only hormone which can influence gambling behaviour (for a review, see [54]). Indeed, in addition to measuring cortisol levels, Coates and Herberg [75] also measured the London day traders’ testosterone levels and found that their morning testosterone levels predicted the day’s profitability. Accordingly, Coates and Herberg found that higher testosterone levels are associated with financial return. Furthermore, in a more recent experiment, Stanton et al. [81] explored the relationship between testosterone and participants’ risk preferences in the Iowa Gambling Task, which is used to measure sensitivity to risk and reward. Specifically, in Stanton et al.’s experiment participants provided a saliva sample for hormonal assessment, and then completed the Iowa Gambling Task in which they had to choose cards from four different decks which differed on their reward and punishment schedules. The results indicated that in both males and females, participants with high testosterone levels took more risks, which in the Iowa Gambling Task is considered to be a disadvantageous decisional pattern, than participants with low testosterone levels. Moreover, there is evidence that males make riskier decisions across a variety of risky behaviours than females [82], including in financial domains [83].

In congruence with evidence that males are generally more risk seeking than females, problem gambling—which is characterised by increased risk-seeking behaviour—tends to be more prevalent among males than females [84,85,86]. Accordingly, as testosterone plays an important role in male sexual development, it has been suggested that individual differences in testosterone levels could explain susceptibility to problem gambling [87]. However, in the first article to explore the link between testosterone and problem gambling, Blanco et al. [88] found that a sample of problem gamblers had testosterone levels were similar in problem gamblers and a healthy control group. Given these results, Blanco et al. [88] concluded that testosterone levels are probably not related to problem gambling. However, more recently Stenstrom and Saad [87] argued that Blanco et al.’s results might have been confounded by a ‘winner-loser effect’ on participants’ testosterone levels (testosterone fluctuates with wins and losses, so differences may not be detected when comparing directly against a healthy sample). Accordingly, in their own review article, Stenstrom and Saad suggested that as testosterone, second-to-fourth digit length ratio (a marker of prenatal testosterone levels) and facial masculinity (a marker of pubertal testosterone) are predictive of financial-risk taking, and that problem gambling is associated with financial risk taking, then it is possible these factors are also predictive of individual problem gambling susceptibility.

### 3.3. The Role of Emotional Activations

In contrast to the normative assumption that ‘rational’ human agents make utility maximising decisions informed solely by psychological computational processing, research from psychology and neuroscience indicate that people’s emotions strongly influence their risky behaviour (see [54]). In particular, Loewenstein and Lerner [89] proposed a distinction between ‘expected’ (or anticipated) and ‘immediate’ emotions. Specifically, expected emotions are those which people forecast to feel after making a choice (it is their prediction about the emotional consequence of decision outcomes—for example, ‘if I choose to gamble and lose, then I will feel terrible’). Accordingly, as expected emotions are relevant to utilitarian predictions and should be factored into utility computations (they are attached to possible decision outcomes), they have been explored predominantly by economists [90,91]. In contrast, psychologists such as Loewenstein [92] have focused on studying the influence of immediate emotions—those which are experience at the time of making a decision and are directly related to the decision context (not attached to possible decision outcomes) [93,94,95,96]. For example, a problem gambler might feel exhilaration at being given an opportunity to gamble. Although, more recently the effects of expected and immediate emotions on people’s behaviour have been explored within a variety of decision-making contexts and tasks (see [54]); specifically, which emotional category (expected or immediate) has a greater influence on people’s risky behaviour. For example, across a series of studies which require participants to engage with economic tasks under conditions of risk, Schlösser et al. [97] provided empirical evidence that people’s decisions were predicted by immediate emotions beyond anticipated emotions, and the subjective probability attached to outcomes.

Furthermore, research exploring the influence of affective states on human behaviour has also examined the influence of positive and negative mood on risk preferences for a review (see [54]). In particular, in relatively recent studies it has been reported that people in a happy mood have higher levels of financial risk tolerance [98], and, relative to a neutral mood, increased risk-seeking behaviour [99]. For instance, Otto et al. [100] found that when incidental outcomes from the environment were more positive than expected (e.g., a better-than-expected sports result), people gamble more. Although, Isen and Patrick [101] found empirical evidence to support their ‘mood maintenance theory’, in which they proposed that people in positive moods avoid risks as they do not want to jeopardise their emotional state. In congruence with these mixed findings, the influence that negative affective states have on risk-taking is also disputed. For instance, given evidence that negative affective states lead people to overestimate the likelihood of experiencing negative future events [102], it has been argued that negative emotions increase risk-averse preferences [103]. However, conversely, some researchers have argued that negative emotions increase risk-taking behaviour [104]. For example, in one of their experiments, Mittal and Ross [104] presented participants with strategic action plans and found that they took more risks after reading a story which induced a negative rather than a positive affective state.

The influence which people’s affective states have on their behaviour have also been explored within specific domains, such as financial [105] and precautionary [106] decision-making [54]. Consistent with the research discussed in the two preceding paragraphs, empirical evidence suggests that in these domains, people’s behaviour is influenced by their emotional state. For instance, Kusev et al. [24] compared participants’ risk preferences for monetary gambles and structurally identical purchase decisions, and found that, relative to monetary gambles, people exaggerated insurance risks. Specifically, participants exaggerated the probability of loss and were more risk-averse when making decisions about more accessible (high-frequency) events than less accessible (low-frequency) events in their memory and hypothetical monetary gambles. Accordingly, the results from Kusev et al. suggest that participants’ experiences with high-frequency events ‘leaked’ into their decisions. Furthermore, in a more recent experiment, Petrova et al. [106] found that buying precautionary insurance is more attractive when a possession (a camera) has an affective (it was a birthday present from your favourite grandfather) rather than neutral (you ordered it via a website) description. Although, interestingly, when the description of the possession was affective, participants’ who engaged in cognitive reappraisal (an emotion regulation strategy) gave responses which were significantly less biased than the participants who did not engage in cognitive reappraisal.

Indeed, given that human behaviour is prone to be influenced by emotions, people often use emotion regulation strategies—such as cognitive reappraisal—to alter their emotional reactions and assure good social and emotional functioning [54,107]. In particular, cognitive reappraisal is a commonly used emotion regulation strategy which is defined as ‘the attempt to reinterpret an emotion-eliciting situation in a way which alters its meaning and changes its emotional impact’ [108] (p. 1). In other words, people reframe the meaning of a situation to alter its emotional impact [54]. However, people with malfunctioning emotion regulation strategies often engage in maladaptive behaviours to escape their negative emotions [109], which can increase the risk that a person will develop symptoms of psychiatric disorders. Accordingly, within the context of problem gambling research, it has been argued that emotional dysregulation (poor emotion regulation)—‘difficulties in identifying, monitoring, evaluating, and/or modulating emotional experience’ [110] (p. 817)—is associated with problem gambling behaviour [110,111]. For example, Williams et al. [111] found that relative to a sample of healthy participants, problem gamblers were less self-aware about their emotional state and reported limited access to effective emotion regulation strategies. Moreover, it has been argued that people with poor emotion regulation strategies can use gambling as a (maladaptive) coping strategy to distract themselves from unpleasant emotions (such those stemming from events in their daily lives), and that this can lead to problem gambling [112,113].

## 4. The Experience of Gambling Behaviour: Dynamic Experience of Events in Time Series

Evidence from behavioural science research reveals that the way decision makers acquire information about decision-making events determines how they respond to it [35,114]. In other words, the experience with decision-making events (their outcome values and likelihoods) informs people’s risky decisions. For example, human decision makers can experience and learn the decision outcomes and likelihood of events by repetitive sampling (dynamic experience), or by having access to available descriptive summary of the outcomes and likelihood of the events [35,114,115]. Accordingly, it could be argued that both problem gamblers and non-problem gamblers usually make decisions based on dynamic experience; they learn about the likelihoods of gambles through repeatedly making gambling decisions and experiencing the outcomes. Hence, gambling behaviour is effectively a dynamic experience of events in time series.

**Figure 2:**
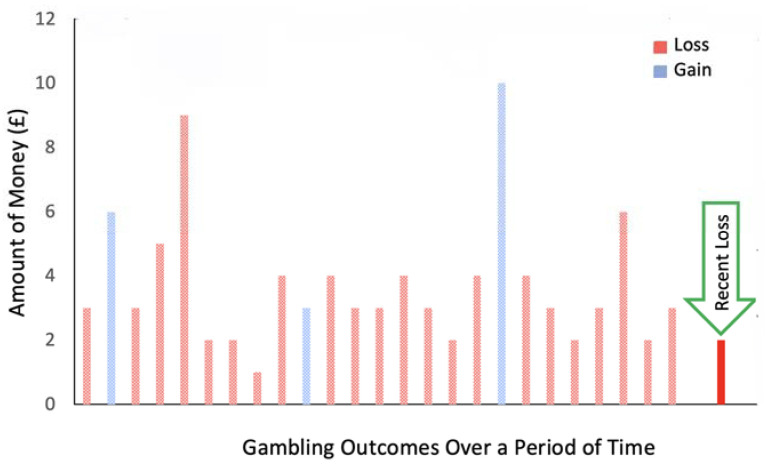
Most recent event (loss) from a dynamicaly experienced sequence of losses and gains.

Furthermore, conducted theoretical and experimental research by Kusev and colleagues [114] demonstrated that when decision-makers experience events in time series, one at a time serially (dynamically), their attention is drawn to each individual event, granting the current (or most recent) event a salient status. Specifically, Kusev et al. [114] found that people tend to anchor on the current (or most recent) event in experienced sequence of events, which has decision-making consequences on how accurately people evaluate past experiences retrospectively. For example, when people attempt to make retrospective evaluations/judgements of their past experiences, these evaluations are informed and altered by the present/most recent event from the dynamically experienced sequences [114] (see Figure 2). Accordingly, it is plausible that the most recent event/gamble, typically resulting in a relatively small loss or rare win, alters the psychological representations of substantial and accumulated loss in the past to a representation of a negligible loss. In other words, people feel better in the present, while they gamble.

This highly adaptive but erroneous decision-making mechanism, where anchoring on the present/most recent event informs the way people feel and judge the past, is likely to influence both problem gamblers and non-problem gamblers. Moreover, it could be argued that the present is the most important and influential psychological state for both problem gamblers and non-problem gamblers. Hence, being in the present and having dynamic experience of gambling events in time series psychologically draws in the decision makers.

## 5. Conclusions

In this article, we explored the underlying psychological constructs and factors associated with problem gambling behaviour. In the exploration we considered the assumption of stability in risky preferences, anticipated and used by both normative and descriptive theories of decision making [1,13]. In contrast, we explored recent evidence that risk preferences are in fact ‘constructed on the fly’ during risk elicitation [14]; risk preferences are sensitive to changes in the decision-making context, in tasks as well as measuring methods. Accordingly, we argued that problem gambling is a multifaceted disorder, which is ‘fuelled on the fly’ by a wide range of contextual and non-contextual influences, including individual differences in biological (hormonal activations) and psychological (personality traits, and emotional activations) factors. We have also proposed that the experience of gambling behaviour in itself is a dynamic experience of events in time series, where both problem gamblers and non-problem gamblers anchor on the most recent event/gamble—typically a small loss or rare win [114]. Accordingly, this anchor informs the way people feel and judge the past by altering the psychological representations of substantial and accumulated loss in the past to a representation of negligible loss. In other words, people feel better in the present, while they gamble.

Given the above, problem gambling researchers, practitioners, as well as policy makers will need to employ multifaceted and holistic approaches to understand problem gambling. In particular, in harmony with existing research efforts [52,53], there is an opportunity to advance the exploration and identification of contextual and task-related factors which fuel problem gambling behaviour (i.e., trigger risk-seeking tendencies). Indeed, a broader understanding of the contextual and task-related factors which increase or decrease the likelihood that a person will engage in problem gambling behaviour will enable policy makers to better regulate the gambling industry, and also to better understand how people could be nudged to make less harmful gambling decisions. Moreover, our proposal that gamblers’ retrospective evaluations/judgements of their past experiences are informed and altered by the most recent event from dynamically experienced sequences is novel, and offers a new avenue of research. Specifically, exploration of this proposal could help researchers, practitioners and policy makers to understand why problem gamblers and non-problem gamblers are drawn to, and subsequently begin to engage in, gambling events.

## Data Availability

Not applicable.
